# Response to Han et al.

**DOI:** 10.14309/ctg.0000000000000449

**Published:** 2022-01-12

**Authors:** Tonya M. Palermo

**Affiliations:** Department of Anesthesiology and Pain Medicine, University of Washington School of Medicine, Seattle, Washington, USA.

Thank you for the opportunity to respond to the letter by Han et al. about our recently published article*, Internet Cognitive-Behavioral Therapy for Painful Chronic Pancreatitis: A Pilot Feasibility Randomized Controlled Trial* (1,2). We appreciate the interest in the article and would like to provide a response to the 3 points raised.

First, Han et al. raise a concern about the period of pain evaluation used at screening vs for outcome assessment. For screening patients into the study, our inclusion criterion required at least moderate pain intensity over the previous month and was intended to recruit individuals into the study who had more severe pain. By contrast, our pain endpoint/outcome used in the pilot trial was the Brief Pain Inventory administered in prospective daily diaries for 7 days at each time point (baseline, 8 weeks, and 3 months). The Brief Pain Inventory is a well-validated measure that has demonstrated responsiveness to change in chronic pain trials (3,4). Measures used for screening and endpoint assessment were not intended to correspond as they served different purposes. However, we wish to highlight that by using prospective electronic diaries for pain assessment, we had the advantage of reducing recall bias and capturing a more accurate estimate of pain intensity and interference. Retrospective single-item measures of average pain intensity, which are most typically used in trials of chronic pain interventions, are limited in accuracy and for describing pain phenotypes.

Our pilot trial was not intended to study pain phenotypes; however, we believe that prospective monitoring of pain experiences in daily diaries could add great value in understanding the pain phenotypes of individuals with chronic pancreatitis (CP). For example, in sickle cell disease, another chronic condition characterized by severe pain, the daily burden of pain in this population has been well documented in the Pain in Sickle Cell Epidemiology Study (PiSCES) (5,6). PiSCES uses pain diaries completed daily over a 6-month period to capture not only the presence, location, intensity, and duration of pain each day but also use of opioids and health care. Because daily diaries allow for the characterization of prospective patterns based on interindividual and intraindividual variability in pain, they have great relevance for better understanding patterns of pain in CP.

The second point raised by Han et al. is that nearly half of the patients enrolled in our pilot trial had comorbid anxiety, depression, and/or sleep disturbance, but we did not report whether these symptoms improved with the intervention. We agree that anxiety, depression, and sleep disturbance are important symptoms in patients with painful CP and contribute to decrements in their quality of life. We plan to conduct a larger trial powered to detect treatment effects across the full range of pain-related outcomes including anxiety, depression, and sleep disturbance as well as other aspects of psychological, social, and physical health in the future. Our pilot feasibility trial was intended to focus on feasibility of the intervention and ensure relevancy and engagement among our target population rather than to demonstrate efficacy.

The third point raised by Han et al. concerns both the Internet cognitive-behavioral therapy group and the control group continuing to receive usual medical care for CP, which may have differed in effectiveness for different individuals in the trial. Usual care could have varied among participants in the pilot trial, and we did not collect detailed information on their concomitant therapies received for pain or disease management during the 8-week intervention. Although it is likely that, with randomization, the groups were balanced on use of concomitant therapies, we agree that future studies should collect this information to better interpret group differences.

## CONFLICTS OF INTEREST

**Guarantor of the article:** Tonya M. Palermo, PhD.

**Specific author contributions:** T.M.P.: prepared the manuscript draft and final draft submitted.

**Financial support:** Research reported in this publication was supported by National institute of Diabetes and Digestive and Kidney Diseases of the National Institutes of Health under award number U01DK108288.

**Potential competing interests:** None to report.
